# Supplementary material for: MD Simulations of Human Sigma‑1 Receptor Trimer Uncover Cholesterol-Dependent Stabilization and Ligand-Specific Dynamics
Source: J Chem Inf Model. 2026 Jun 1;66(12):7296–306. doi: 10.1021/acs.jcim.6c01322 (PMC13292196; doi:10.1021/acs.jcim.6c01322)
Supplement: Supplementary file 1 [file ci6c01322_si_001.pdf]

Supporting Information

MD simulations of Human Sigma1 Receptor Trimer Uncovers Cholesterol Dependent Stabilization and Ligand Specific Dynamics

*Vittoria Nanna[1] ‡, Costanza Paternoster[3] ‡, Alessio Bartocci[3], Dritan Siliqi[1], Domenico Alberga[1], Carmen Abate[4], Gianluca Lattanzi[5,6] *, Giuseppe Felice Mangiatordi[1] **

[1]Istituto di Cristallografia, Consiglio Nazionale delle Ricerche (CNR), via Amendola 122/O, Bari, 70126, Italy
[2]Department of Experimental Oncology, Istituto Europeo di Oncologia, IRCCS, Milano, Italy
[3]Dipartimento di Biotecnologie Mediche e Medicina Traslazionale, Università degli Studi di Milano, via Fratelli Cervi, 93, Segrate, 20054, Italy.
[4]Department of Pharmacy – Pharmaceutical Sciences, University of Bari "Aldo Moro", Via E. Orabona, 4, I-70125 Bari, Italy
[5]Dipartimento di Fisica, Università di Trento, via Sommarive 14, Trento, 38123, Italy.
[6]INFN-TIFPA, Trento Institute for Fundamental Physics and Applications, via Sommarive, 14, Trento, 38123, Italy.

‡These authors contributed equally to this work.

*Correspondence: gianluca.lattanzi@unitn.it and giuseppefelice.mangiatordi@cnr.it

List of contents

Supplementary Figures

Figure S1: Structure of cholesterol (CHOL), phosphatidylcholine (POPC) and phosphatidylethanolamine (POPE) – the lipids that compose the membrane in which the S1R receptor is embedded. Relevant atom nomenclature is reported. The black dashed line represents the vector used to calculate the lipid tilt angles.

Figure S2: Root-mean square deviation (RMSD) of the protein Cα atoms over the course of the simulations for the whole protein (A, B) and the core (residues 36–223) (C, D). In all cases, RMSD was computed relative to the first frame after alignment on the Cα atoms of the entire protein. Panels A and C show the S1R–Hal and S1R–PnT systems in a pure POPC membrane, whereas panels B and D show the corresponding systems in the MAM-mimicking POPC/POPE/CHOL membrane.

Figure S3: Boxplot with quartiles, median and outliers of the lipid-averaged area per lipid (APL) (A) and bilayer thickness (B), computed across all replicates for the four simulated systems. The POPC membrane (in red and light blue) exhibits a higher APL and a correspondingly lower bilayer thickness compared with the POPC/POPE/CHOL system (in pink and light green).

Figure S4: Analysis of the membrane curvature induced by S1R–PnT and S1R–Hal in the MAM-mimicking (A) and in POPC (B) membrane outer leaflet, in each simulation replicate (n=1, 2, 3). Top row: 2D maps of the lipids' head (P atom) z-coordinate variation from lowest z-coordinate value (Z). Middle row: 2D maps of the membrane mean curvature (H). Bottom column: 2D maps of the membrane gaussian curvature (K). (C) Geometric interpretation of the curvature values H and K.

Figure S5: S1R receptor modulates local membrane organization. (A) Ordered lipids are observed in the crystal structures 6dk1 (left) and 6djz (right) at the junction of the luminal core and the α1 helix (shown in yellow sticks, oxygen atoms in red). Protein ligands (+)-pentazocine and haloperidol are shown in blue and pink, respectively. (B) Steroid-binding regions of S1R are highlighted on the S1R-progesterone structure (8w4b): two steroid binding domain-like regions (SBDLI and SBLII) and CARC-like motif.

Supplementary Tables

Table S1: Membrane composition for S1R-Hal and S1R-PnT systems.

Table S2: Simulation box details.

Table S3: Summary of simulations performed in this work.

Table S4: Gromacs simulation parameters employed for trajectories production.

Table S5: Averaged area per lipid (APL) in the upper and lower leaflet of the membrane computed across all replicates for the four simulated systems.

Figure S1

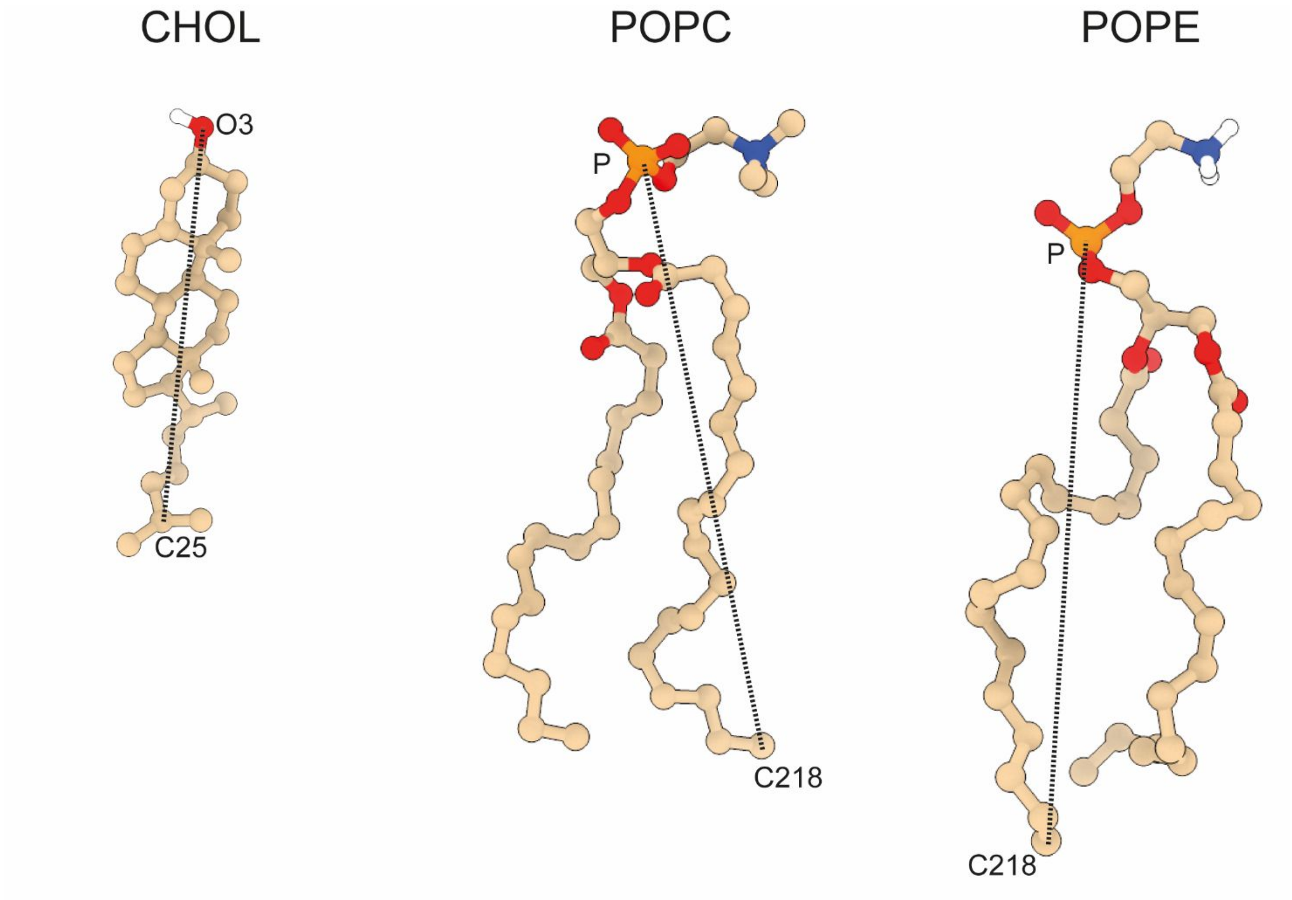

Figure S2.

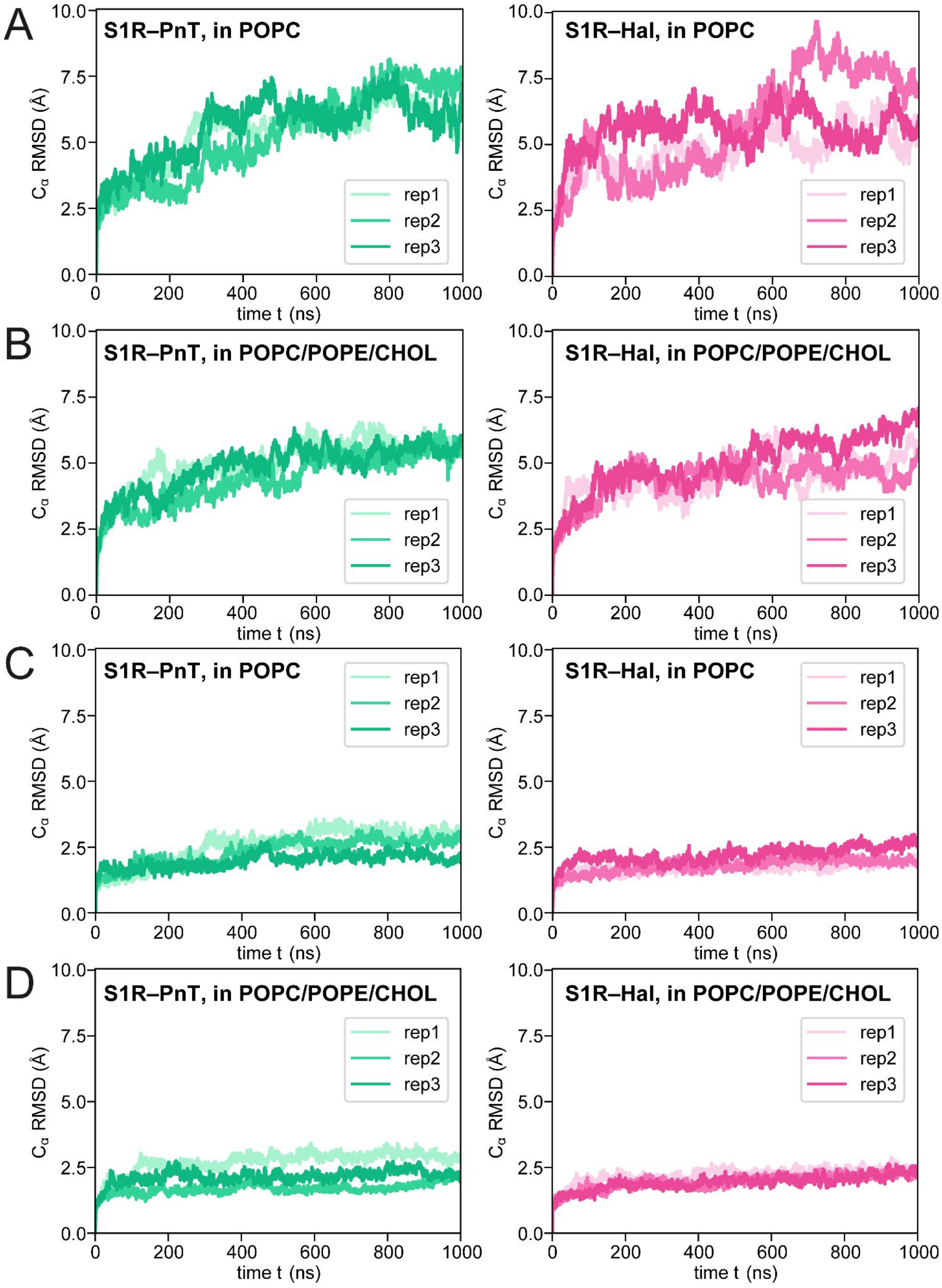
A
S1R–PnT, in POPC
S1R–Hal, in POPC
B
S1R–PnT, in POPC/POPE/CHOL
S1R–Hal, in POPC/POPE/CHOL
C
S1R–PnT, in POPC
S1R–Hal, in POPC
D
S1R–PnT, in POPC/POPE/CHOL
S1R–Hal, in POPC/POPE/CHOL
rep1
rep2
rep3
Cα RMSD (Å)
time t (ns)
0.0
2.5
5.0
7.5
10.0
0
200
400
600
800
1000

Figure S3.

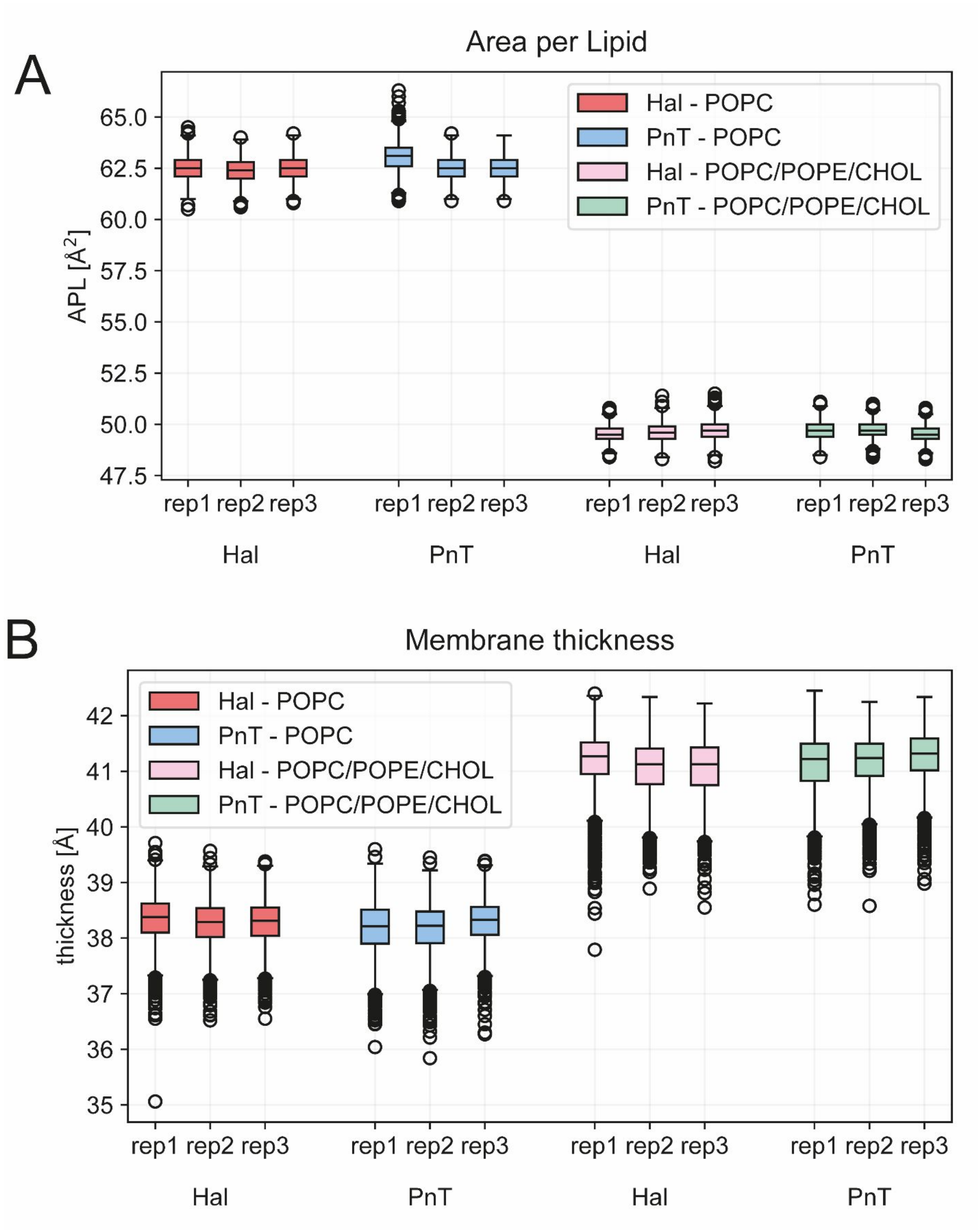

A
Area per Lipid
APL [Å^2]
65.0
62.5
60.0
57.5
55.0
52.5
50.0
47.5
Hal - POPC
PnT - POPC
Hal - POPC/POPE/CHOL
PnT - POPC/POPE/CHOL
rep1 rep2 rep3
rep1 rep2 rep3
rep1 rep2 rep3
rep1 rep2 rep3
Hal
PnT
Hal
PnT
B
Membrane thickness
thickness [Å]
42
41
40
39
38
37
36
35
Hal - POPC
PnT - POPC
Hal - POPC/POPE/CHOL
PnT - POPC/POPE/CHOL
rep1 rep2 rep3
rep1 rep2 rep3
rep1 rep2 rep3
rep1 rep2 rep3
Hal
PnT
Hal
PnT

Figure S4.

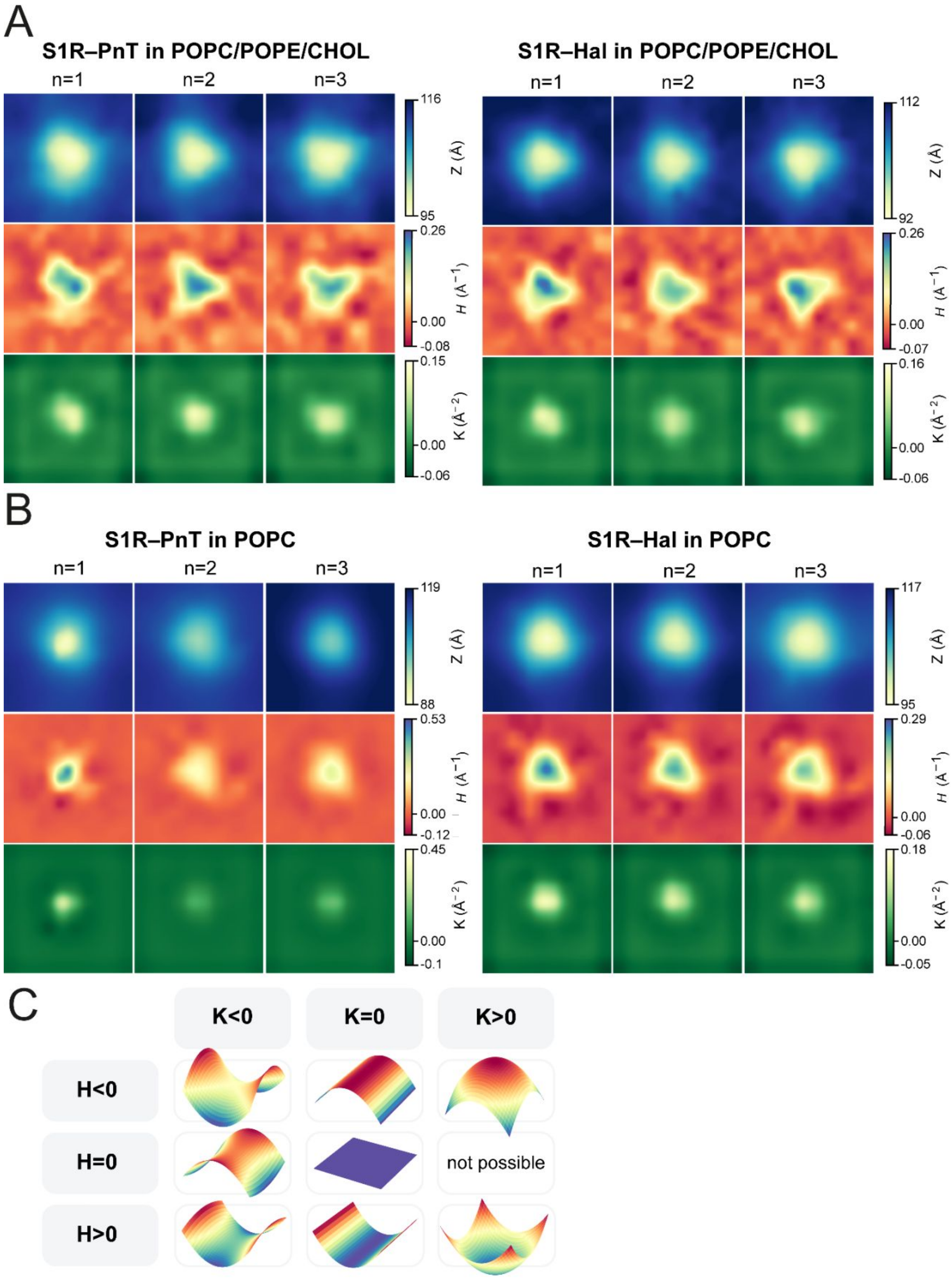
A
S1R–PnT in POPC/POPE/CHOL
S1R–Hal in POPC/POPE/CHOL
n=1
n=2
n=3
Z (Å)
H (Å^{-1})
K (Å^{-2})
116
95
0.26
0.00
-0.08
0.15
0.00
-0.06
112
92
0.26
0.00
-0.07
0.16
0.00
-0.06
B
S1R–PnT in POPC
S1R–Hal in POPC
119
88
0.53
0.00
-0.12
0.45
0.00
-0.1
117
95
0.29
0.00
-0.06
0.18
0.00
-0.05
C
K<0
K=0
K>0
H<0
H=0
H>0
not possible

Figure S5.

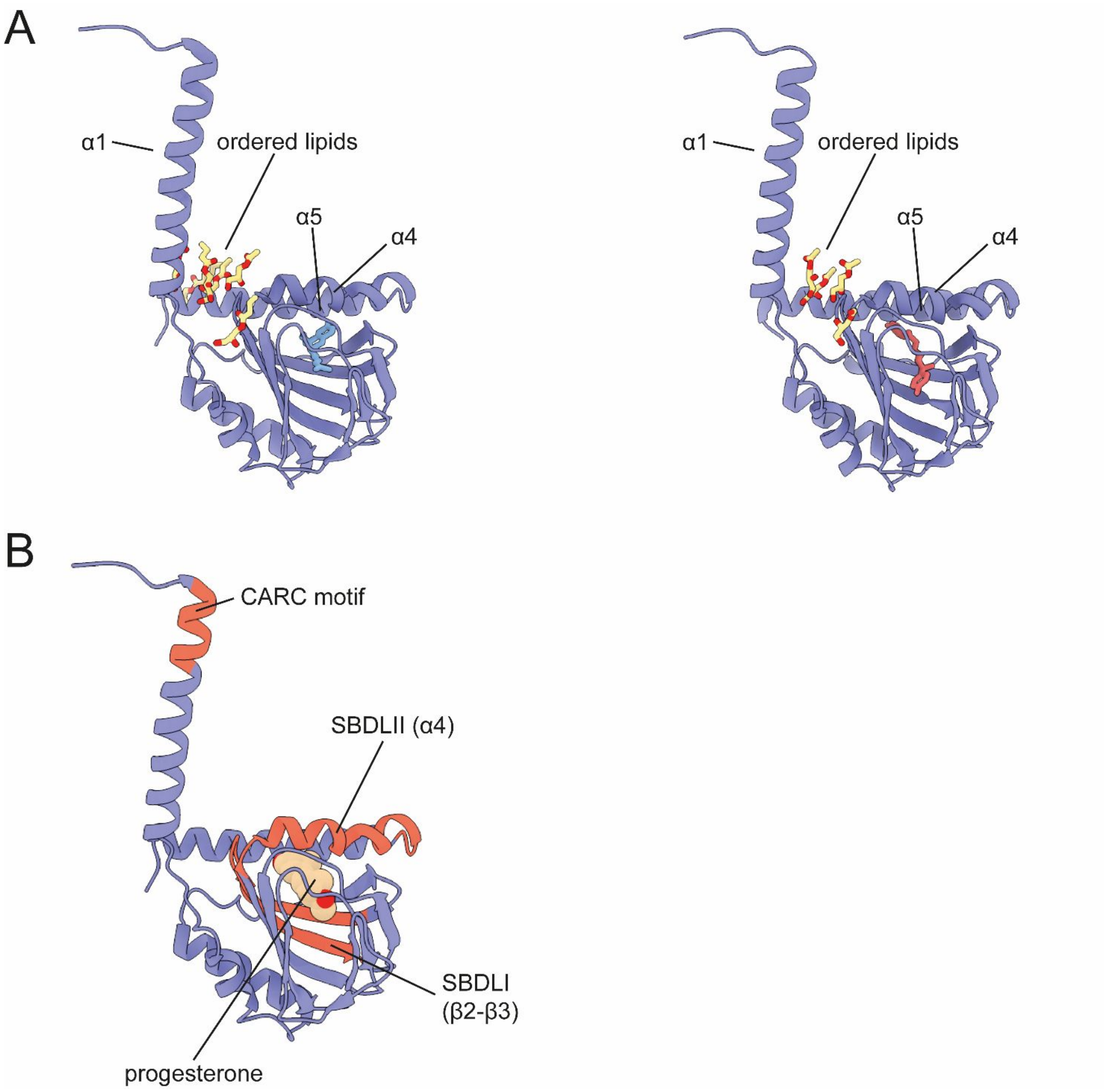

Table S1.

**System**	**Leaflet**	**POPC**	**CHOL**	**POPE**	**TOT LIPIDS**
S1R-Hal in POPC	Inner	326	0	0	326
	Outer	365	0	0	365
S1R-PnT in POPC	Inner	325	0	0	325
	Outer	364	0	0	364
S1R-Hal in MAM-like membrane	Inner	224	88	88	400
	Outer	252	99	99	450
S1R-PnT in MAM-like membrane	Inner	224	88	88	400
	Outer	252	99	99	450

Table S2.

	S1R-Hal in POPC	S1R-PnT in POPC	S1R-Hal in MAM-like membrane	S1R-**PnT** in MAM-like membrane
Water mol	78884	81341	85084	88011
Lipid mol	691	689	850	850
Na^+ mol	227	234	246	255
Cl^- mol	217	224	236	245
Tot atoms	340507	347618	367548	376341
Initial box dimensions (x, y, z) [nm]	16.0,16.0,14.3	16.0,16.0,14.6	16.6,16.6,14.3	16.6,16.6,14.6

Table S3.

System	Membrane	Replicates	Simulation length (per replicate)	Total simulation time
S1R-Hal	POPC	3	1 µs	3 µs
S1R-PnT	POPC	3	1 µs	3 µs
S1R-Hal	MAM-like membrane	3	1 µs	3 µs
S1R-PnT	MAM-like membrane	3	1 µs	3 µs
**TOTAL**		**12**		**12 µs**

Table S4.

Parameter	Value
integrator	md
dt	0.002
nstxtcout	25000
nstcalcenergy	100
nstenergy	1000
nstlog	1000
cutoff-scheme	Verlet
verlet-buffer-tolerance	-1
nstlist	50
rlist	1.33
vdwtype	Cut-off
vdw-modifier	Force-switch
rvdw_switch	1.0
rvdw	1.2
coulombtype	PME
rcoulomb	1.2
pbc	xyz
tcoupl	v-rescale
tc_grps	SOLU MEMB SOLV
tau_t	1.0 1.0 1.0
ref_t	310 310 310
nsttcouple	50
pcoupl	C-rescale
pcoupltype	semiisotropic
tau_p	5.0
compressibility	4.5e-5 4.5e-5
ref_p	1.0 1.0
nstpcouple	50
constraints	h-bonds
constraint_algorithm	LINCS
continuation	yes
nstcomm	100
comm_mode	linear
comm_grps	SOLU_MEMB SOLV

Table S5.

**Hal - POPC**	
Inner leaflet	63.06 ± 0.71 $Å^2$
Outer leaflet	61.95 ± 0.58 $Å^2$
**PnT - POPC**	
Inner leaflet	63.10 ± 0.73 $Å^2$
Outer leaflet	62.43 ± 0.81$Å^2$
**Hal - POPC/POPE/CHOL**	
Inner leaflet	50.13 ± 0.58 $Å^2$
Outer leaflet	49.25 ± 0.40 $Å^2$
**PnT - POPC/POPE/CHOL**	
Inner leaflet	50.11 ± 0.52 $Å^2$
Outer leaflet	49.18 ± 0.38 $Å^2$
